# Differential expression profiles and functional analysis of long non-coding RNAs in calcific aortic valve disease

**DOI:** 10.1186/s12872-023-03311-x

**Published:** 2023-06-27

**Authors:** Guang-Yuan Song, Xu-Nan Guo, Jing Yao, Zhi-Nan Lu, Jia-Hong Xie, Fang wu, Jing He, Zhao-Lin Fu, Jie Han

**Affiliations:** 1 Interventional Center of Valvular Heart Disease, Beijing Anzhen Hospital Affiliated to Capital Medical University, Beijing, China; 2 Department of Cardiac Surgery, Beijing Anzhen Hospital Affiliated to Capital Medical University, Beijing, China

**Keywords:** Calcific Aortic Valve Disease, Cis-regulation, Trans-regulation, WGCNA, Long noncoding RNAs, Competitive endogenous RNAs

## Abstract

**Aim:**

To evaluate the expression profile of long non-coding RNAs (lncRNAs) in calcific aortic valve disease (CAVD) and explore their potential mechanism of action.

**Methods:**

The gene expression profiles (GSE153555, GSE148219, GSE199718) were downloaded from the Gene Expression Omnibus (GEO) database and FastQC was run for quality control checks. After filtering and classifying candidate lncRNAs by differentially expressed genes (DEGs) and weighted co-expression networks (WGCNA) in GSE153555, we predicted the potential *cis-* or *trans-*regulatory target genes of differentially expressed lncRNAs (DELs) by using FEELnc and established the competitive endogenous RNA (ceRNA) network by miRanda, more over functional enrichment was analyzed using the ClusterProfiler package in R Bioconductor. The hub *cis-* or *trans-*regulatory genes were verified in GSE148219 and GSE199718 respectively.

**Results:**

There were 340 up-regulated lncRNAs identified in AS group compared with the control group (|log_2_Fold Change| ≥ 1.0 and P_adj_ ≤ 0.05), and 460 down-regulated lncRNAs. Based on target gene prediction and co-expression network construction, twelve Long non-coding RNAs (*CDKN2B-AS1, AC244453.2, APCDD1L-DT, SLC12A5-AS1, TGFB3, AC243829.4, MIR4435-2HG, FAM225A, BHLHE40-AS1, LINC01614, AL356417.2, LINC01150*) were identified as the hub *cis-* or *trans-*regulatory genes in the pathogenesis of CAVD which were validated in GSE148219 and GSE19971. Additionally, we found that *MIR4435-2HG* was the top hub *trans-*acting lncRNA which also plays a crucial role by ceRNA pattern.

**Conclusion:**

LncRNAs may play an important role in CAVD and may provide a new perspective on the pathogenesis, diagnosis, and treatment of this disease. Further studies are required to illuminate the underlying mechanisms and provide potential therapeutic targets.

**Supplementary Information:**

The online version contains supplementary material available at 10.1186/s12872-023-03311-x.

## Introduction

Calcific aortic valve disease (CAVD) is the most common cause of aortic stenosis (AS) and has become an increasing economic and health burden for human populations [1]. Although previous views believed that CAVD was a passive calcium deposition process, recent studies have found that it is an actively regulated process that involves valve endothelial disruption, lipid infiltration, immune cell infiltration, extracellular matrix remodeling, apoptosis, and deranged phospho-calcium metabolism [2]. However, the pathophysiological process of CAVD remains unclear and the treatment strategies for CAVD mainly rely on surgery or transcatheter aortic valve replacement (TAVR). Thus, identification of key genes and pathways is crucial for exploring the molecular mechanisms of CAVD, to find molecular targets for early diagnosis, prevention, and specific treatment of AS.

Non-coding RNAs (ncRNAs), which constitute nearly 98% of the human genome, have always been regarded as junk RNA but were confirmed to participate in various pathophysiological processes [3]. Based on their length, ncRNA can be classified as small ncRNAs and long ncRNAs. LncRNAs are ncRNAs longer than 200 nt which participate in cellular processes at epigenetic, transcriptional, post-transcriptional levels and other modes of gene regulation via chromatin modification、*cis*- or *trans-r*egulation [4] or competitive endogenous RNAs [5]. Evidence suggests that dysregulated lncRNAs play a vital role in cardiovascular diseases, such as atherosclerosis, myocardial infarction, cardiac fibrosis [6], and CAVD [7–9]. However, few studies have focused on the *cis*- or *trans*-regulation and ceRNAs regulation of lncRNAs in CAVD.

With the advancements of gene chips, high-throughput sequencing and single-cell sequencing, bioinformatics techniques have become instrumental in studying diseases at the molecular level. In contrast to traditional methods to identify DEGs, the weighted co-expression networks (WGCNA) [10] are used to construct different modules by describing transcriptome expression patterns, which can also elucidate the correlation of mRNA and lncRNA. Multiple bioinformatics methods can provide key genes and pathways in the process of calcification, which will help discover new directions for interventions and therapeutic targets for CAVD.

In this study, DEGs and DELs were first screened based on GSE153555 and the results were validated in GSE148219 and GSE199718. Subsequently, we predicted the potential *cis*- and *trans-r*egulatory target genes of DELs by using FEELnc software and established the ceRNA network by miRanda. Additionally, integrated bioinformatics analyses, including Principal component analysis (PCA), Weighted correlation network analysis, Gene Ontology term analysis and ROC curve analysis were performed by R version 4.1.3.

## Results

### Overview of long noncoding RNAs expressed in aortic valve stenosis

**Fig. 1:**
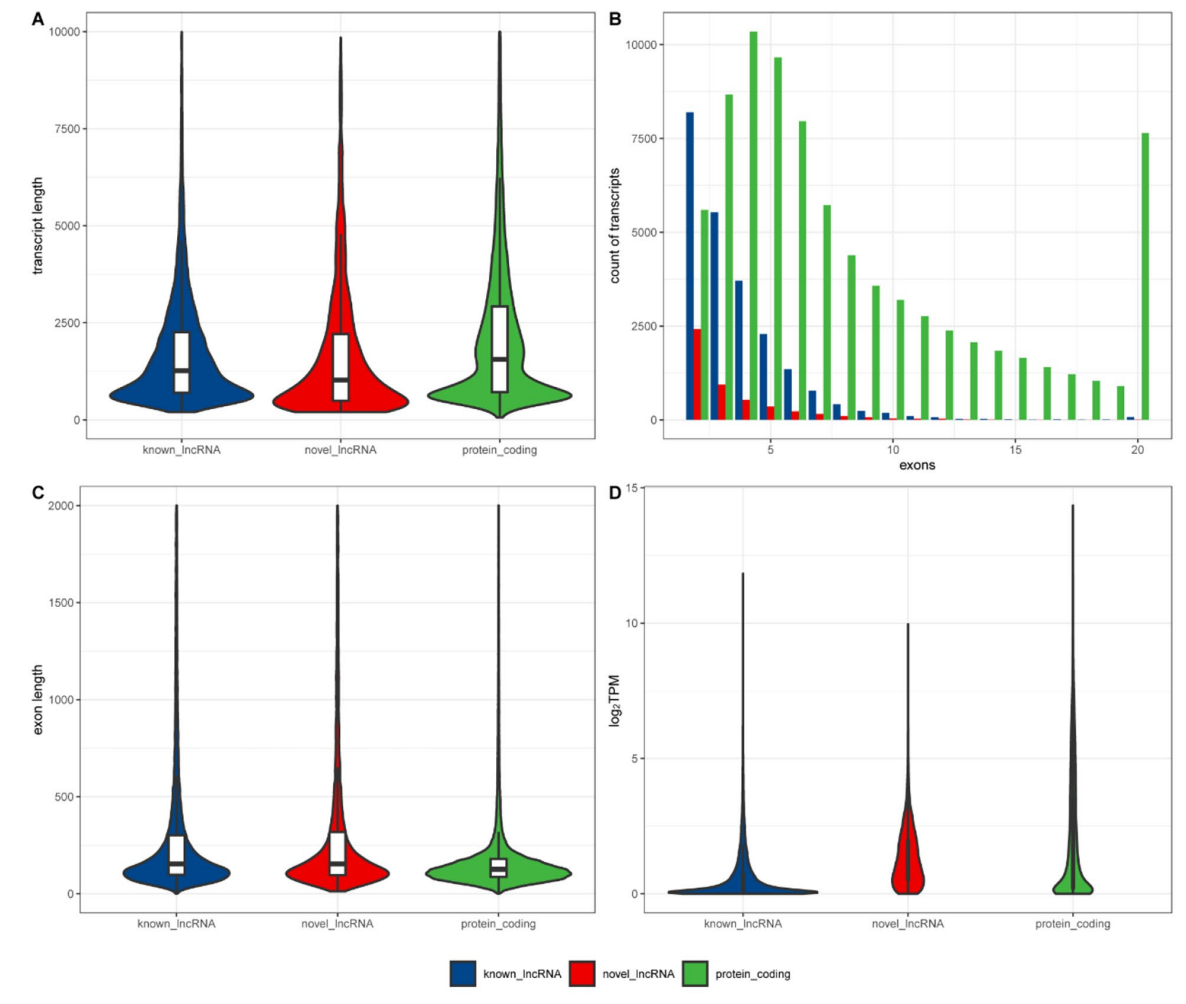
Characterization of lncRNAs compared with protein-coding genes Comparison of (**A**) transcript length; (**B**) numbers of exons; (**C**) exon length; (**D**) expression level

To investigate the transcriptome pattern in aortic valves of healthy and aortic stenosis (AS) patients, RNA-seq data were downloaded from GSE153555 [11]. To validate the expression and diagnostic values of hub *cis-* or *trans-*regulatory genes in the pathogenesis of CAVD, RNA-seq data of GSE148219 and GSE199718 were downloaded. The sample characteristics of the above datasets are presented in Table 1. In GSE153555, by performing *in silico* prediction of lncRNAs, we identified 27,960 lncRNA transcripts of 11,996 lncRNA genes. By comparison with GENCODE annotation version 31, we detected 4926 novel lncRNA transcripts, and the average transcript length of the novel lncRNAs (1774 nt) was similar to that of known lncRNAs (1814 nt) and shorter than that of protein-coding genes (2275 nt) (Fig. 1A). At the exonic level, although novel lncRNAs (at an average of 3.72 per transcript) have similar counts of exons to known lncRNAs (at an average of 3.48 per transcript), less than protein-coding genes (at an average of 9.03 per transcript) (Fig. 1B), the average exon length of novel lncRNAs (503 nt) and known lncRNAs (388 nt) were longer than protein-coding genes (252 nt) (Fig. 1C). In the normalized read count expression level (log_2_TPM), the average values of known lncRNAs, novel lncRNAs, and protein-coding genes were 0.593, 1.35, and 2.09, respectively (Fig. 1D).

**Table 1:** The sample characteristics of GEO datasets

Characteristics		GSE153555 N = 20			GSE148219 N = 20			GSE199718 N = 22		
		Control	CAVD	P	Control	CAVD	P	Control	CAVD	P
Sex	Male	2 (20.00%)	4 (40.00%)	0.6285	7 (87.50%)	10 (83.33%)	> 0.9999	10 (83.33%)	9 (90.00%)	> 0.9999
	Female	8 (80.00%)	6 (60.00%)		1 (12.50%)	2 (16.67%)		2 (16.67%)	1 (10.00%)	
Age		37.23 ± 19.02	73.80 ± 2.93	0.001	37.38 ± 12.94	66.75 ± 9.35	< 0.0001	49.50 ± 10.83	59.90 ± 4.74	0.0023
Tissue	BAV	0 (0.00)	2 (20.00%)	0.4737	0 (0.00)	5 (41.67%)	0.0547	0 (0.00)	3 (41.67%)	0.0022
	TAV	10 (100.00%)	8 (80.00%)		8 (100.00%)	7 (58.33%)		12 (100.00%)	10 (33.33%)	

Independent-samples T test was used to compare continuous data. Pearson’s chi-squared test was used to compare the categorical data

### Divergent expression patterns of protein-coding genes and lncRNA genes

**Fig. 2:**
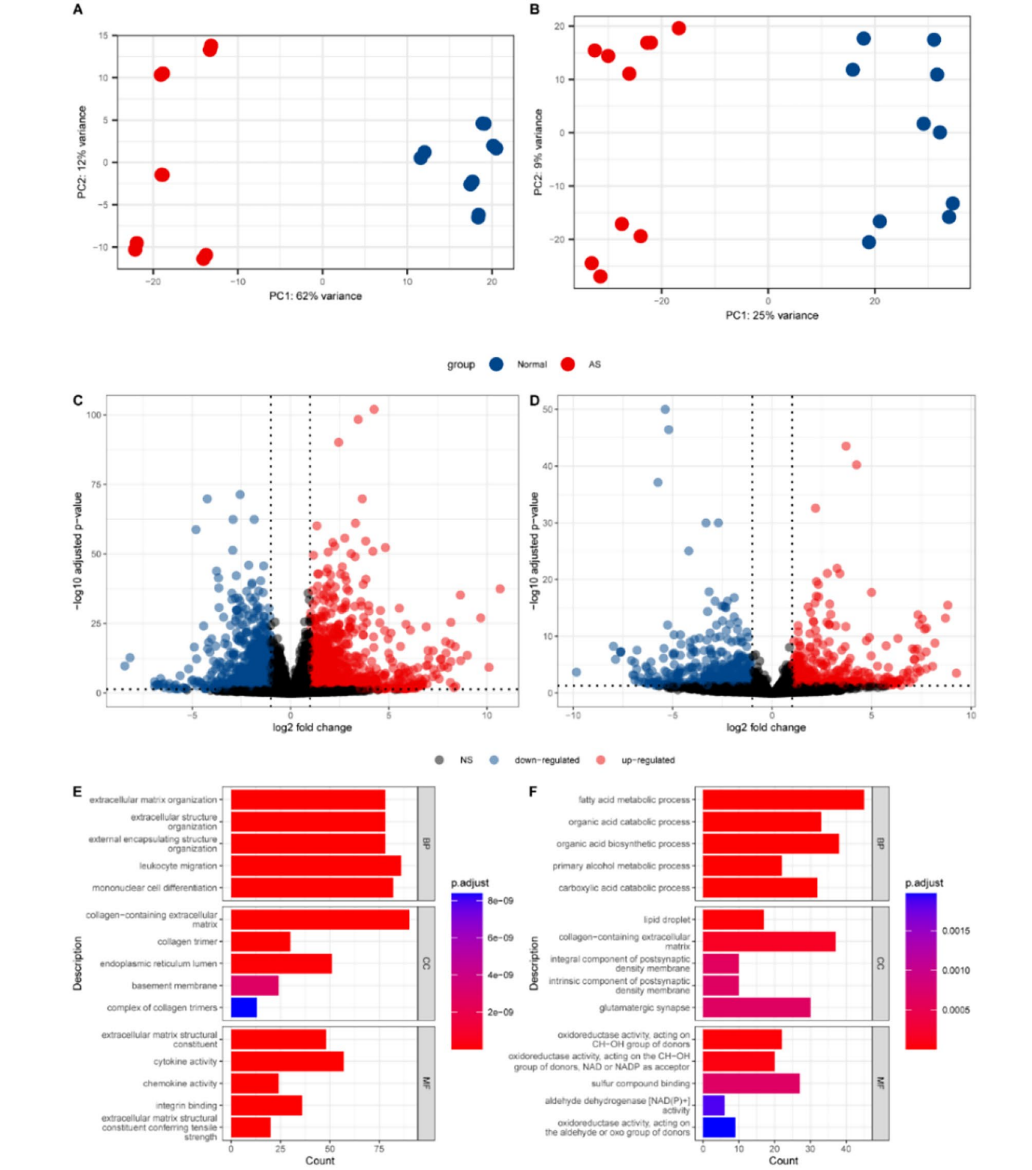
DEGs and DELs are identified in differential expression analysis PCA is based on (**A**) mRNA and (**B**) lncRNA. Volcano plots of (**C**) DEGs and (**D**) DELs. GO analysis of (**E**) upregulated DEGs and (**F**) downregulated DEGs

Principal component analysis (PCA) showed obvious discrimination between the AS and normal groups in both protein-coding genes (Fig. 2A) and lncRNAs (Fig. 2B). By performing differential gene expression analyses for mRNA and lncRNA individually, a total of 1904 protein-coding genes and 800 lncRNA genes were detected to be differentially expressed (|log_2_Fold Change| ≥ 1.0 and *p*_adj_ ≤ 0.05), with 1119 upregulated, 785 downregulated protein-coding genes (Fig. 2C) and 340 upregulated, 460 downregulated lncRNA genes (Fig. 2D) in the AS group compared with the control group (Table 2, Table S1, Table S2).

**Table 2:** TOP 3 differentially expressed mRNAs and lncRNAs in GSE153555

RNA Symbol/ID	RNA Type	Base Mean	log_2_FoldChange	P value	Adjusted P value	Regulation
*CBLN4*	mRNA	155.38	10.66	1.11E-40	3.70E-38	Up
*MMP13*	mRNA	1071.31	10.1	3.05E-11	6.03E-10	Up
*LY6H*	mRNA	78.58	9.68	7.65E-30	1.03E-27	Up
*ZNF804B*	mRNA	30.16	-8.44	8.47E-12	1.81E-10	Down
*OR8D4*	mRNA	30.72	-8.17	6.15E-15	1.92E-13	Down
*TRIML2*	mRNA	10.59	-6.93	1.27E-05	9.34E-05	Down
MSTRG.26,064	lncRNA	49.97	9.26	1.47E-05	3.20E-04	Up
MSTRG.41,599	lncRNA	37.04	8.83	1.12E-18	3.32E-16	Up
MSTRG.61,873	lncRNA	34.13	8.71	3.13E-16	6.36E-14	Up
MSTRG.39,342	lncRNA	87.05	-9.83	8.83E-06	2.11E-04	Down
MSTRG.88,982	lncRNA	24.04	-7.98	7.06E-11	5.70E-09	Down
MSTRG.75,410	lncRNA	22.28	-7.87	2.61E-08	1.18E-06	Down

To further clarify the functional differences of protein-coding genes between the two groups, we performed Gene Ontology (GO) enrichment analysis. The top enrichment terms of cellular component (CC) ranked by *p*_adj_ showed that the products of both the activated genes and suppressed genes were associated with the extracellular matrix (Fig. 2E, F). The top enrichment terms of Biological Process (BP) for activated genes contribute to extracellular matrix modulation and immune response, and the suppressed genes lead to metabolic dysregulation (Fig. 2E, F), which were reported in previous research [11, 12].

### Weighted correlation network analysis showed the key module associated with AS

**Fig. 3:**
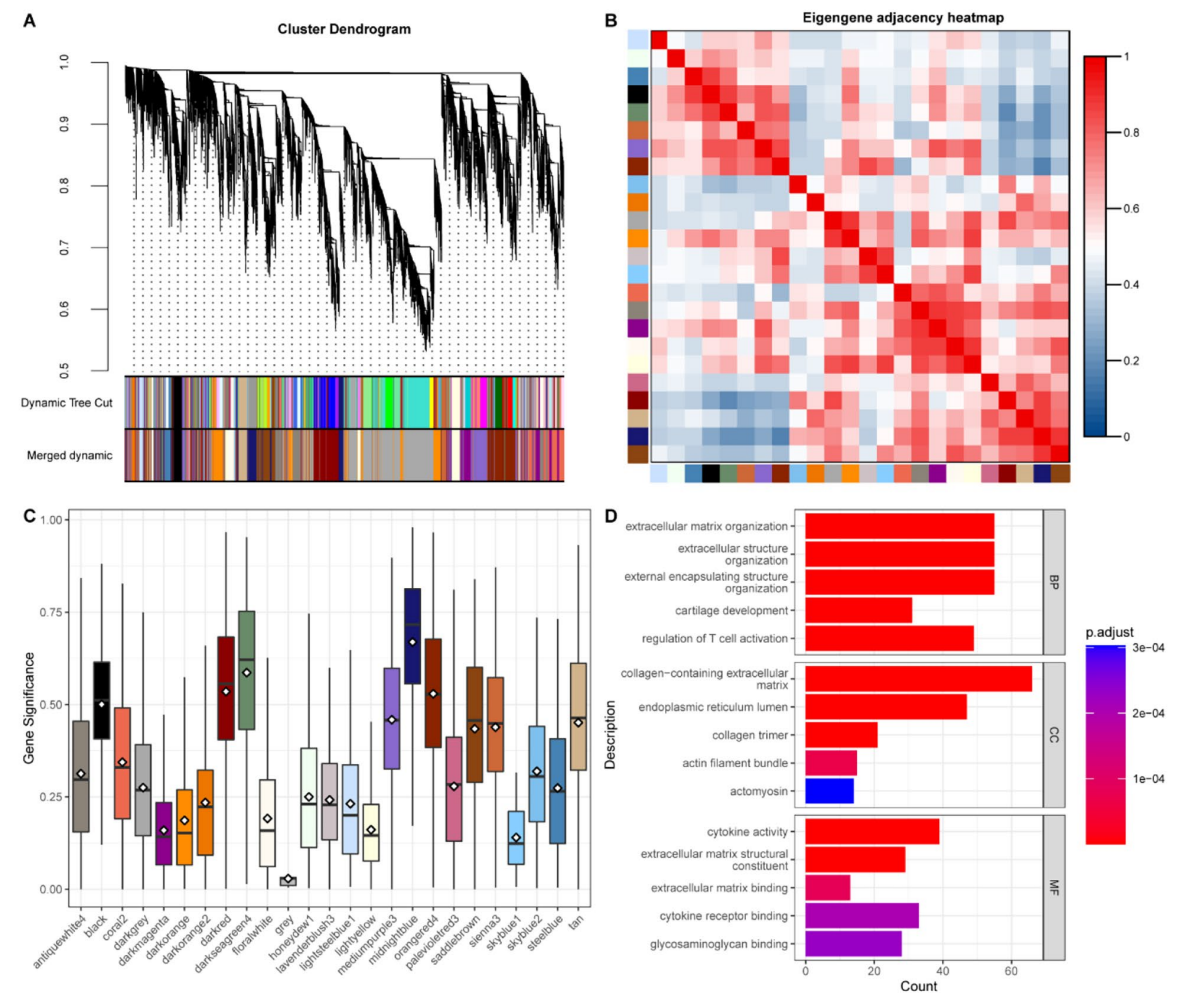
Identifying the key module associated with AS (**A**) Total genes were clustered into 25 modules, and each module is marked with one color. (**B**) Heatmap of distances showing obvious discrimination among modules. (**C**) Module gene significance in relation to AS. (**D**) GO analysis of midnightblue module

To identify the hub mRNAs and lncRNAs associated with AS, we constructed an unsigned weighted correlation network analysis (WGCNA) network. Hierarchical clustering analysis was conducted based on unsigned weighted correlation before segmenting according to to set criteria to obtain gene modules and merged modules too close as measured by the correlation of their eigengenes (Fig. 3A). A total of 25 modules were detected, with an average size of 936.4 genes (including protein-coding and lncRNA genes), ranging from 65 to 5,287 genes, whose relationship is shown in a heatmap (Fig. 3B).

By calculating the gene significance (GS) associated with the disease and control groups, the midnightblue module (including 1,194 genes, with 891 protein-coding genes and 303 lncRNA genes) showed the highest correlation to AS (Fig. 3C), which was considered the key functional module.

Considering the expression status of genes in each module, the midnightblue module contained 22.3% of all DEGs (424 in 1904), which was ranked as the highest proportion among all the modules, and was 47.6% of protein-coding genes in the midnightblue module, which was ranked second among all modules (Table 3), indicating that the midnightblue module reflected the expression difference between the two modules. Furthermore, we performed GO enrichment of the midnightblue module and found a strong association with extracellular matrix organization (Fig. 3D), which was similar to GO enrichment of upregulated DEGs (Fig. 2E), indicating the functional representation of the midnightblue module and establishing the importance of the midnightblue module.

**Table 3:** Genes in WGCNA-identified modules

module	mRNA	lncRNA	DEG	DEL	DEG/mRNA	DEL/lncRNA	DEG/all DEG	DEL/all DEL
midnightblue	891	303	424	73	47.6%	24.1%	22.3%	9.1%
darkred	1450	756	366	136	25.2%	18.0%	19.2%	17.0%
orangered4	1030	662	357	81	34.7%	12.2%	18.8%	10.1%
mediumpurple3	726	729	196	57	27.0%	7.8%	10.3%	7.1%
saddlebrown	773	351	172	44	22.3%	12.5%	9.0%	5.5%
tan	358	137	117	27	32.7%	19.7%	6.1%	3.4%
darkseagreen4	121	73	63	7	52.1%	9.6%	3.3%	0.9%
coral2	835	246	60	17	7.2%	6.9%	3.2%	2.1%
black	149	437	39	28	26.2%	6.4%	2.0%	3.5%
sienna3	85	163	32	9	37.6%	5.5%	1.7%	1.1%
steelblue	187	411	21	19	11.2%	4.6%	1.1%	2.4%
darkgrey	4427	860	13	15	0.3%	1.7%	0.7%	1.9%
skyblue2	37	43	8	3	21.6%	7.0%	0.4%	0.4%
palevioletred3	58	84	8	3	13.8%	3.6%	0.4%	0.4%
lightsteelblue1	71	128	8	5	11.3%	3.9%	0.4%	0.6%
darkorange2	64	111	7	6	10.9%	5.4%	0.4%	0.8%
antiquewhite4	1305	290	5	8	0.4%	2.8%	0.3%	1.0%
lavenderblush3	47	82	3	2	6.4%	2.4%	0.2%	0.3%
honeydew1	28	98	2	4	7.1%	4.1%	0.1%	0.5%
darkorange	1521	555	2	5	0.1%	0.9%	0.1%	0.6%
floralwhite	274	51	1	0	0.4%	0.0%	0.1%	0.0%
darkmagenta	1146	317	0	0	0.0%	0.0%	0.0%	0.0%
grey	2	6	0	1	0.0%	16.7%	0.0%	0.1%
lightyellow	694	172	0	1	0.0%	0.6%	0.0%	0.1%
skyblue1	31	34	0	0	0.0%	0.0%	0.0%	0.0%

### ***Cis***-regulation functions of lncRNAs in the key module associated with AS

By analyzing genes in the midnightblue module, we found that 24.1% of lncRNAs were differentially expressed among all 303 lncRNA genes, which was the highest proportion among the modules, and was 9.1% of all DELs (Table 3), which was ranked third among the modules, showing the potential important regulatory function of lncRNAs in this module. Previous studies have shown that lncRNAs can regulate the expression of target genes and participate in functional regulation by *cis*- or *trans*-regulation [4].

**Fig. 4:**
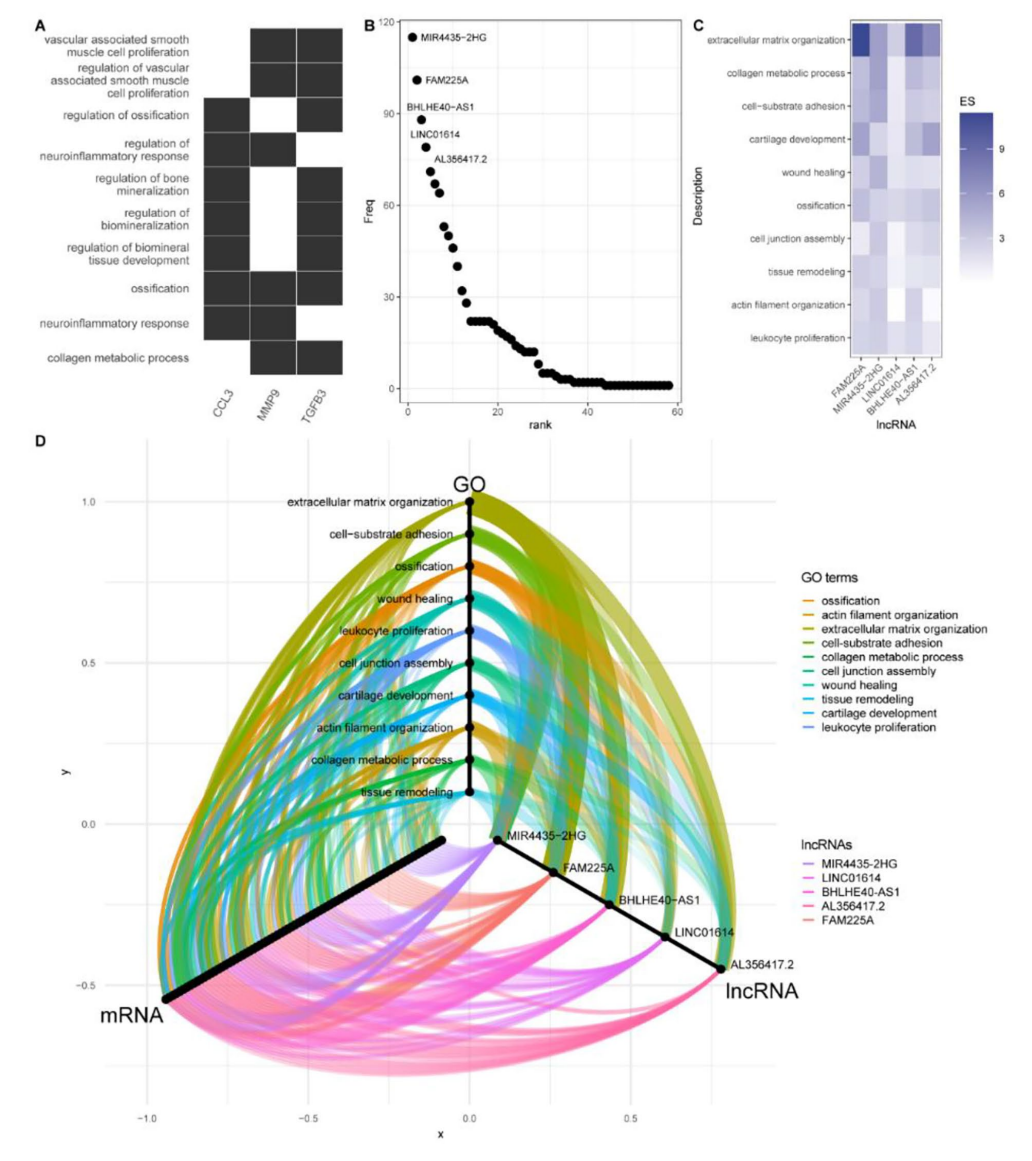
*Cis*- and *trans*-regulatory functions of lncRNAs in the midnightblue module (**A**) Heat plot of GO enrichment of *cis*-mRNA in midnightblue module. The black cells in the heat map represent the GO terms that were enriched by cis-mRNAs, while the white cells represent the GO terms that were not enriched by the cis-mRNAs; (**B**) number of *trans-*regulated protein-coding genes by lncRNAs in midnightblue module; (**C**) enrichment score (ES) of GO enrichment of mRNAs *trans-*regulated by lncRNAs; (**D**) association of lncRNAs, their coexpressed mRNAs, and GO enrichment terms. Width of edges between GO terms and lncRNAs related to the number of potential mRNAs *trans-*regulated by lncRNAs enriched in GO terms

First, we predicted the potential *cis*-regulatory target genes of DELs by using FEELnc software, which was used to detect coding genes adjacent to candidate lncRNAs. We obtained 38,364 pairs of colocalized lncRNA genes with the best match protein-coding genes, which included 23 protein-coding genes and 23 lncRNA genes in the midnightblue module. Nine pairs of mRNA‒lncRNA were also detected to be differentially expressed, which were the key *cis-* regulatory lncRNAs and mRNAs (key *cis-* lncRNA and *cis-* mRNA) (Table 4). In particular, the key *cis-* mRNAs *MMP9*, *CCL3*, and *TGFB3* are involved in ossification (Fig. 4A). *MMP9*, which plays a certain role in cardiovascular remodeling [13], is *cis-* regulated by its antisense lncRNA *SLC12A5-AS1*. *TGFB3*, which can drive fibrotic disease pathogenesis [14], is positively regulated by its lncRNA isoforms. *CCL3* is involved in inflammation and ossification and is regulated by *AC243829.4*.

**Table 4:** Key cis-mRNAs and lncRNAs in midnightblue module

mRNA ID	mRNA Symbol	lncRNA ID	lncRNA Symbol	Direction	Type	Subtype
ENSG00000147889	*CDKN2A*	MSTRG.82,657	*CDKN2B-AS1*	antisense	genic	overlapping
ENSG00000198019	*FCGR1B*	MSTRG.4218	*AC244453.2*	antisense	genic	overlapping
ENSG00000198768	*APCDD1L*	MSTRG.50,482	*APCDD1L-DT*	antisense	intergenic	divergent
ENSG00000277632	*CCL3*	MSTRG.33,890	*AC243829.4*	antisense	genic	overlapping
ENSG00000100985	*MMP9*	MSTRG.50,021	*SLC12A5-AS1*	antisense	genic	overlapping
ENSG00000130592	*LSP1*	MSTRG.11,770	*LINC01150*	sense	intergenic	same_strand
ENSG00000119699	*TGFB3*	MSTRG.24,884	*TGFB3*	sense	intergenic	same_strand
ENSG00000134107	*BHLHE40*	MSTRG.53,614	*BHLHE40-AS1*	antisense	genic	overlapping
ENSG00000164694	*FNDC1*	MSTRG.73,401	*AL356417.2*	antisense	genic	overlapping

### ***Trans***-regulation functions of lncRNAs in the key module associated with AS

In addition, lncRNAs can influence the expression of target genes by *trans*-regulation. By performing coexpression analysis, we detected 192,291 pairs of highly coexpressed mRNA‒lncRNA genes (Pearson’s *r* > 0.9 and *p* < 0.01), including 1,163 pairs consisting of 397 mRNAs and 58 lncRNAs in the midnightblue module. Of the 58 coexpressed lncRNAs, several lncRNAs were coexpressed with quantities of protein-coding genes, with *MIR4435-2HG* having the most numerous coexpressed protein-coding genes at 115 (Fig. 4B). The lncRNAs with the top 5 degrees were considered hub lncRNA genes in the midnightblue module. To further investigate the *trans-*regulatory functions of those hub-lncRNAs, we performed GO enrichment for their *trans-*acting protein-coding genes. Hub *trans-*regulatory genes besides *LINC01614* showed a strong relation with extracellular matrix organization (Fig. 4C, D, Supplementary Figure S1) which may work through *trans-*regulate *COL3A1, MMP9, OLFML2B, COL27A1, NPNT etc.*. Among lncRNAs associated with extracellular matrix organization, *FAM225A*, *BHLHE40-AS1*, and *AL356417.2* showed a stronger relationship with ossification and cartilage development, which may lead to calcification by *trans-*regulate *BMP3, RUNX2, CCN1, MMP13, CCN3 etc.*. It is noteworthy that *MIR4435-2HG* may *trans-*regulate *SERPINH1, COL1A1, COL1A2, P3H4, ADAMTS14 etc.* to regulate collagen metabolism in CAVD. *Trans-*acting protein-coding genes of *LINC01614* showed less relation to the extracellular matrix but a stronger association with leukocyte proliferation (*BCL6, CD28, CD38, IL34, BTN3A1 etc.*) and cell junction assembly (*TBX5, ICAM5, IRX3, AGRN, NEGR1 etc.*) than other genes (Fig. 4C, D, Supplementary Figure S1).

### ceRNA network of lncRNAs in the key module associated with AS

**Fig. 5:**
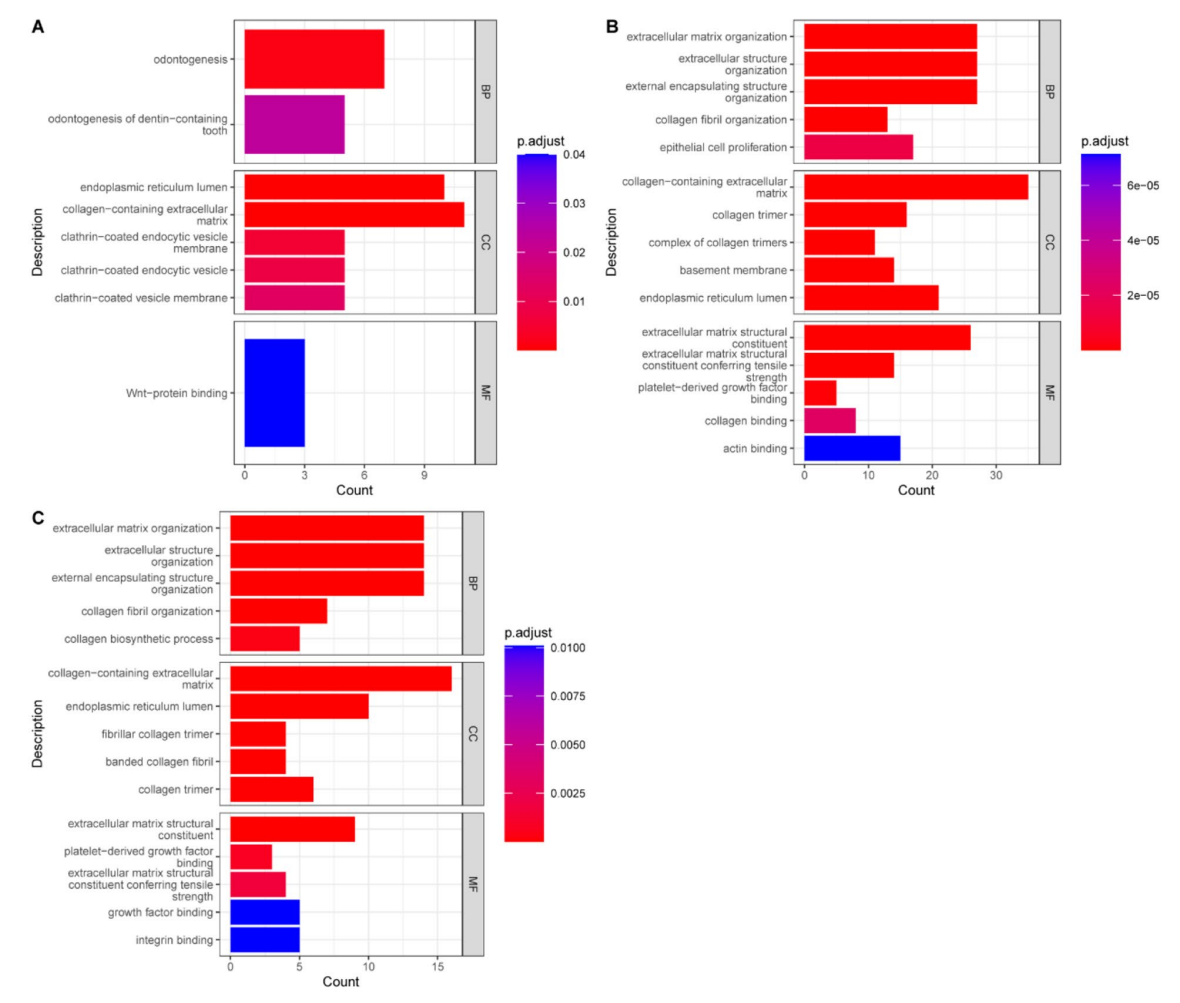
Potential functions of lncRNAs through ceRNA mechanisms in midnightblue module GO enrichment analysis for mRNAs regulated by (**A**) *MIR4435-2HG*; (**B**) *FAM225A*; and (**C**) *BHLHE40-AS1* through a ceRNA mechanism

Interestingly, we found that *MIR4435-2HG*, which was the host gene of *MIR4435* and was reported to be a key regulatory lncRNA in multiple diseases through the molecular mechanism of competitive endogenous RNA networks [15, 16], was the top hub *trans-*acting lncRNA, indicating the potential roles of lncRNA‒miRNA interactions in AS pathophysiology. To reveal the ceRNA in AS, we predicted the targets of all human miRNAs at mRNAs and lncRNAs by miRanda, and the mRNAs-lncRNA pairs that had at least 1 common miRNA binding site and were also detected to be highly coexpressed were considered potential ceRNA mRNA‒lncRNA pairs. Finally, we constructed a ceRNA network consisting of 1217 DEGs, 273 DELs, and 2628 miRNAs, including 230 protein-coding genes and 32 lncRNA genes in the midnightblue module. Among all lncRNAs in the midnightblue module, *AL589743.7* had the largest number of miRNA binding sites with 1412 miRNAs, while *MIR4435-2HG* (with 1412 miRNAs), *CYTOR* (with 1214 miRNAs), *FAM225A* (with 713 miRNAs), and *BHLHE40-AS1* (with 591 miRNAs) also ranked at the top 5 in midnightblue module. Furthermore, *FAM225A* can regulate 77 mRNAs through the ceRNA mechanism, while *MIR4435-2HG*, *LINC01614*, *BHLHE40-AS1*, and *AL356417.2* can regulate the expression of 70, 68, 63, 61 mRNAs in midnightblue module. Combining the relationship between lncRNAs and miRNAs with lncRNAs with mRNAs, *MIR4435-2HG* had 9,268 potential lncRNA‒miRNA-mRNA axes, ranking the highest in the midnightblue module. To clarify the potential mechanism through miRNA, we performed GO analysis of potential ceRNA target mRNAs of *MIR4435-2HG*, *FAM225A*, and *BHLHE40-AS1*. While *MIR4435-2HG* showed a relationship with odontogenesis and endocytic vesicles, which are associated with ossification, *FAM225A* and *BHLHE40-AS1* both showed a relationship with collagen-associated extracellular matrix and structural constituents conferring tensile strength (Fig. 5).

### ROC curves analysis of hub genes

**Fig. 6:**
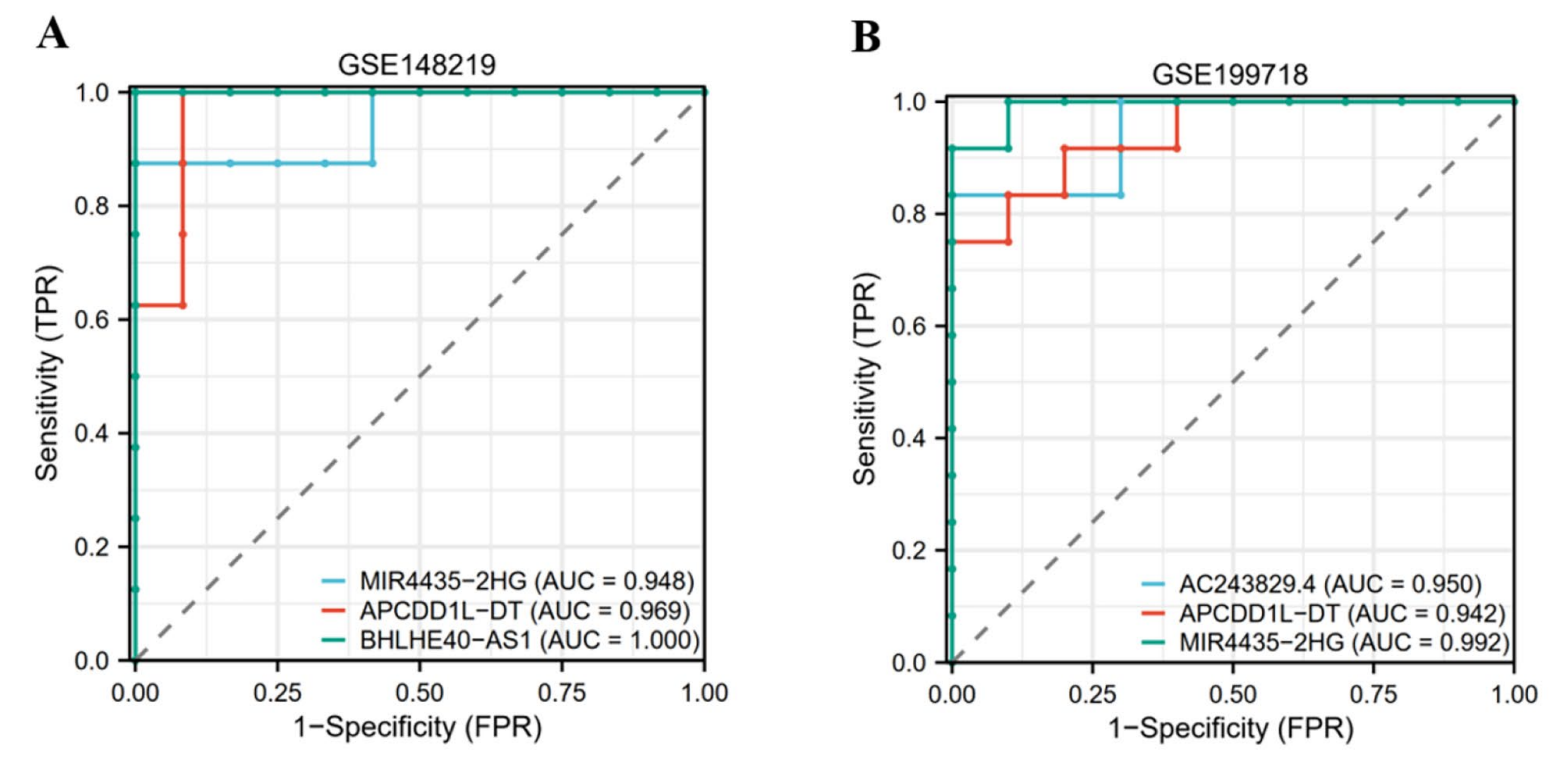
ROC curves analysis of hub *cis-* or *trans-*regulatory genes AUC based on each of the top 3 hub genes in (**A**) GSE148219 (**B**) GSE199718

We constructed ROC curves of each hub *cis-* or *trans-*regulatory genes separately and found that their area under ROC curves (AUC) of *CDKN2B-AS1, AC244453.2, APCDD1L-DT, SLC12A5-AS1, TGFB3, AC243829.4, MIR4435-2HG, FAM225A, BHLHE40-AS1, LINC01614, AL356417.2, LINC01150* were all higher than 0.7 in GSE148219 (Fig. 6A, Figure S2). In GSE199718, AUC were higher than 0.7 respectively (Fig. 6B, Figure S2), expect for *LINC01150* (AUC = 0.575) and *CDKN2B-AS1* (AUC = 0.625). These indicate that these twelve genes have a good ability to discriminate between calcified and normal valves.

## Materials and methods

### Genome-wide RNA-seq data of mRNAs and lncRNAs in AS

The RNA expression dataset of GSE153555 was derived from the study of Greene CL et al. [11], which collected 210.6 Gb data from 10 samples of AS and 10 control samples. Transcriptome sequencing data for GSE199718 and GSE148219 were derived from the study of Cheng S et al. [17]and MacGrogan D et al. [18]. To ensure the reliability of raw reads and suitability for downstream analysis, FastQC was run for quality control checks [19], and the sequences of poor quality were trimmed and filtered by trim_galore 0.6.6 [20] to obtain clean reads. The resulting reads were mapped against the reference genome GRCh38 downloaded from GENCODE by STAR version 2.7.6a [21].

### Bioinformatics identification of lncRNAs

Transcripts of 20 samples were assembled individually by StringTie v2.2.1 [22], and then transcripts of all 20 samples were combined by the merge parameter of StringTie into a nonredundant transcript set. Candidate lncRNA transcripts were filtered and classified by the FEElnc [23] pipeline to obtain the high-confidence set of lncRNA transcripts. The high-confidence set of lncRNA transcripts was compared with GENCODE version 31 by gffcompare v0.11.2 [24], and transcripts with class codes of “i”, “j”, “o”, “u”, and “x” were considered as *novo* transcripts.

### Differential expression analysis of mRNAs and lncRNAs

mRNA and lncRNA abundance was quantified using StringTie [22]. The TPM matrix generated by StringTie was used to summarize the lncRNA and mRNA expression levels. Gene differential expression analysis was performed by DESeq2 v1.34.0 [25] in R/Bioconductor (R version 4.1.0). Gene ontology enrichment analysis was performed by clusterProfiler v4.2.0 [26] using the database package org.Hs.eg.db v3.14.0.

### WGCNA

The unsigned WGCNA network was constructed by WGCNA v1.71 [10] using an experienced soft power of 6 to obtain gene modules. Modules too close as measured by the correlation of their eigengenes were merged using a cutoff height of 0.20. Gene significances were calculated for the trait of the AS group compared to the healthy control, and the module that had the highest average GS of genes was identified as the key module associated with AS.

### ***Cis***- and ***trans*** -regulation analysis

FEELnc [23] was used to search for the coding genes near the confirmed lncRNAs (upstream and downstream 10–100 kb), taking the parameter “isBEST = 1” for *cis*-regulation analysis. Hmisc 4.7-0 was used to calculate the Pearson’s correlation *r* of lncRNAs and mRNAs, and mRNAs with |*r*| > 0.9 and *p* value < 0.01 were viewed as coexpressed with their lncRNAs.

### Prediction of miRNA targets

miRNA sequences were retrieved from microrna.org. Miranda v3.3a [27] was used to predict the binding sites of miRNA against the full-length lncRNAs and 3’UTR of mRNAs. The lncRNA-mRNA pairs were predicted to have binding sites for the same miRNA and also to be coexpressed were considered potential ceRNA pairs.

### Data visualization

All visualizations were performed in R version 4.1.3. Graphs were plotted using the ggplot2 v3.3.5 package [28] or the preimplemented function “plot” unless otherwise noted. Heatmap was drawn using the pheatmap v1.0.12 package (https://cran.r-project.org/web/packages/ pheatmap/index.html). Network graph was drawn using the igraph v1.3.1 (https://cran.r-project.org/web/packages/igraph/citation.html), tidygraph v1.2.0 (https://cran.r-project.org/web/packages/tidygraph/index.html), and ggraph v2.0.5 packages (https://cran.r-project.org/web/packages/ggraph/index.html).

## Discussion

CAVD is the most common valvular heart disease that frequently leads to aortic stenosis and heart failure in developed countries. Up to now, the etiology and pathogenesis of CAVD are still undetermined. In recent years, notable progress can be observed in miRNA-based therapies. Nevertheless, the complex regulatory mechanisms restrict the applications of LncRNA in clinical. Based on their position relative to protein-coding genes, LncRNA can be classified as sense lncRNA, antisense lncRNA, intronic lncRNA, bidirectional lncRNA and intergenic lncRNA. *Cis-*regulation [29], a form of transcriptional activation and expression regulation of adjacent protein-coding gene mRNAs by lncRNAs (distance lower than 10 kb), make up an important part of the lncRNA regulation network together with *trans-*regulation [4] and ceRNA [30]. As demonstrated in our results, obvious discrimination can be detected between the AS and normal groups in both protein-coding genes and lncRNAs. Thus, a comprehensive understanding of the lncRNA-mediated network is essential for uncovering the pathophysiological processes of CAVD and identifying potential therapeutic targets.

In this study, we utilized bioinformatics techniques to investigate the potential relationships between lncRNA and mRNA in aortic calcification. Deriving lncRNA function from mRNAs is an important research strategy. To better understand the mechanisms of target mRNAs in DELs, the FEELnc and miRanda were used to build *cis-* / *trans-*regulation and lncRNA-miRNA-mRNA regulatory networks. The GO enrichment analysis based on the DEGs and lncRNA-mediated mRNAs between AS and control are mainly associated with the extracellular matrix, immune response and metabolic dysregulation which were consistent with current studies.

As the most important risk factors for cardiovascular disease, metabolic dysregulation especially lipid metabolisms have been expected to become new therapeutic targets for aortic stenosis. Our study showed that the GO enrichment analysis of downregulated DEGs had a tight link with fatty acid and organic acid metabolisms. Although GWAS research from over 114,000 UK Biobank participants did not prove the protection role of circulating polyunsaturated fatty acids in cardiovascular disease [31]. However, Gonzalo Artiach et al. demonstrated that Omega-3 polyunsaturated fatty acids decrease aortic valve disease through the resolvin E1 and chemR23 axis [32]. Therefore, fatty acids, as the substrates for various lipids, may become the novel treatment method for CAVD through alleviation of inflammation progress [33]. CAVD is regarded as an active inflammatory process, similar to atherosclerosis, involving both the adaptive and the innate immune systems [34]. The upregulated DEGs were also enriched in the immune response but the key gene and potentially mediated lncRNAs still need subsequent discovery and verification.

By FEELnc, we predicted the key *cis-*mRNAs *MMP9*, *CCL3*, and *TGFB3* which are both classic ossification genes. The research of key *cis-*lncRNA *SLC12A5-AS1*, *AC243829.4* and *TGFB3* mainly concentrated in cancer [35, 36] and immune respone [37]. It is interesting to note that the lncRNA *AC243829.4* is the ferroptosis-related lncRNA. Despite still being in its infancy, ferroptosis showed a wide range of perspectives in valve calcification [38, 39].

As research progresses, the ECM is not only considered to be passive mechanical support of the aortic leaflets but also a complex cellular microenvironment that is closely related to the development of CAVD [40]. The ECM of the normal aortic valve is composed of elastin, collagen and proteoglycans which are mainly secreted by aortic valve interstitial cells [41]. Excess provisional extracellular matrix is also regarded as a common factor in Bicuspid Aortic Valve formation [42]. Although the relationship between ECM and non-coding RNA was determined in different diseases [43], few studies have focused on lncRNAs regulatorily dysfunctional ECM in CAVD. As shown in the result, both upregulated DEGs and DELs were strongly association with extracellular matrix organization. By FEELnc, we predicted that the long noncoding gene *FAM225A, AL356417.2* and *BHLHE40-AS1* can regulate mRNA by *trans-*regulation function or ceRNA which existing research focuses on cancer and immune disease [44–46].

Different from the above ECM regulator lncRNA, GO enrichment for the *trans-*acting protein-coding genes *LINC01614* showed a relationship with phosphate ion transmembrane transport. Calcium phosphate deposition is the characteristic of vascular calcification and its transporters were strictly controlled by *eNPP1, 5NT, ENT1, Pit-2* and *ANK* [47]. Dysregulated phosphate metabolism enhances the osteoblast gene and promotes mineralization [48]. According to existing literature, *LINC01614* can promote pancreatic cancer progression by *WNT/β‑catenin* signaling which is the crucial pathway in aortic valve calcification [49]. While expression of the *WNT/β‑catenin* signaling pathway can also be against phosphate-induced calcification [50]. Therefore we assume that *LINC01614* can regulate phosphate metabolism by *trans-*regulation through *WNT/β‑catenin* signaling. However, the hypothesis still needs further experimental verification.

We finally focused our attention on the *MIR4435-2HG*, also known as LncRNA *AWPPH*, which ranked the highest in the midnightblue module ceRNA networks and had the most numerous coexpressed protein-coding genes by *trans-*regulation. As the potential pan-cancer biomarker [51] and hub gene of cardiovascular disease [52, 53], *MIR4435-2HG* also participates in process of osteogenesis and osteolysis disease [16]. Except for ECM, *MIR4435-2HG* showed a stronger relationship with collagen metabolism and *Wnt-*protein binding. Collagen, as the main ingredient of ECM, secretion by dysfunctional cells promotes calcification further by *BMPs* (bone morphogenetic proteins) and *WNT/β‑catenin* signaling. Although there is no relevant research, collagen metabolism may be considered as a new therapeutic target for the early treatment of CAVD. Notably, Xiaofang et al. demonstrated that the *MIR4435-2HG* was significantly increased in plasma samples of periodontitis patients which was remarkably decreased after treatment [54]. To date, several reports have described the relationship between periodontal and aortic calcification [55] or carotid artery calcification [56], but the association between periodontal and CAVD is still controversial. As a result, *MIR4435-2HG* may become a potential breakthrough in understanding the common pathways between periodontal and aortic calcification. In summary, *MIR4435-2HG*, which can regulate multiple osteogenesis genes through various pathways, will occupy an important position in the diagnosis and treatment of CAVD.

## Conclusion

Via DEGs、DELs and WGCNA, we established an omnifarious lncRNA regulatory network in CAVD and identified 12 hub LncRNA which throughout the pathological process of extracellular matrix, immune response and metabolic dysregulation in CAVD. These crucial genes and networks provide future trends for basic research and new directions for interventions and therapeutic targets. It should be noted that our study only focused on bioinformatics analysis. The next step in future research experiments is required to clarify the result in vitro and in vivo.

## Electronic Supplementary Material

Below is the link to the electronic supplementary material.

Supplementary Material 1

Supplementary Material 2

Supplementary Material 3

Supplementary Material 4

## Data Availability

The datasets analysed during the current study are available in the NCBI-GEO database (https://www.ncbi.nlm.nih.gov/geo/). The data that support the findings of this study are available from the corresponding author, Guang-Yuan Song or Jie Han, upon reasonable request.
