# Supplementary material for: Differential expression profiles and functional analysis of long non-coding RNAs in calcific aortic valve disease
Source: BMC Cardiovasc Disord. 2023 Jun 27;23:326. doi: 10.1186/s12872-023-03311-x (PMC10294343; doi:10.1186/s12872-023-03311-x)
Supplement: Supplementary file 1 — Supplementary Material 1 [file 12872_2023_3311_MOESM1_ESM.pdf]

Differential Expression Profiles and Functional Analysis of Long Non-coding RNAs in Calcific Aortic Valve Disease

Guang-Yuan Song[1]*,Xu-Nan Guo[1],Jing Yao[1],Zhi-Nan Lu[1],Jia-Hong Xie[1],Fang wu[2],Jing He[1],Zhao-Lin Fu[1],Jie Han[2]*

[1]Interventional Center of Valvular Heart Disease, Beijing Anzhen Hospital Affiliated to Capital Medical University, Beijing, China

[2]Department of Cardiac Surgery, Beijing Anzhen Hospital Affiliated to Capital Medical University, Beijing, China

[*] Co-Correspondence author: Guang-Yuan Song, email: songgy_anzhen@vip.163.com, Jie Han, email:drhanjie@163.com

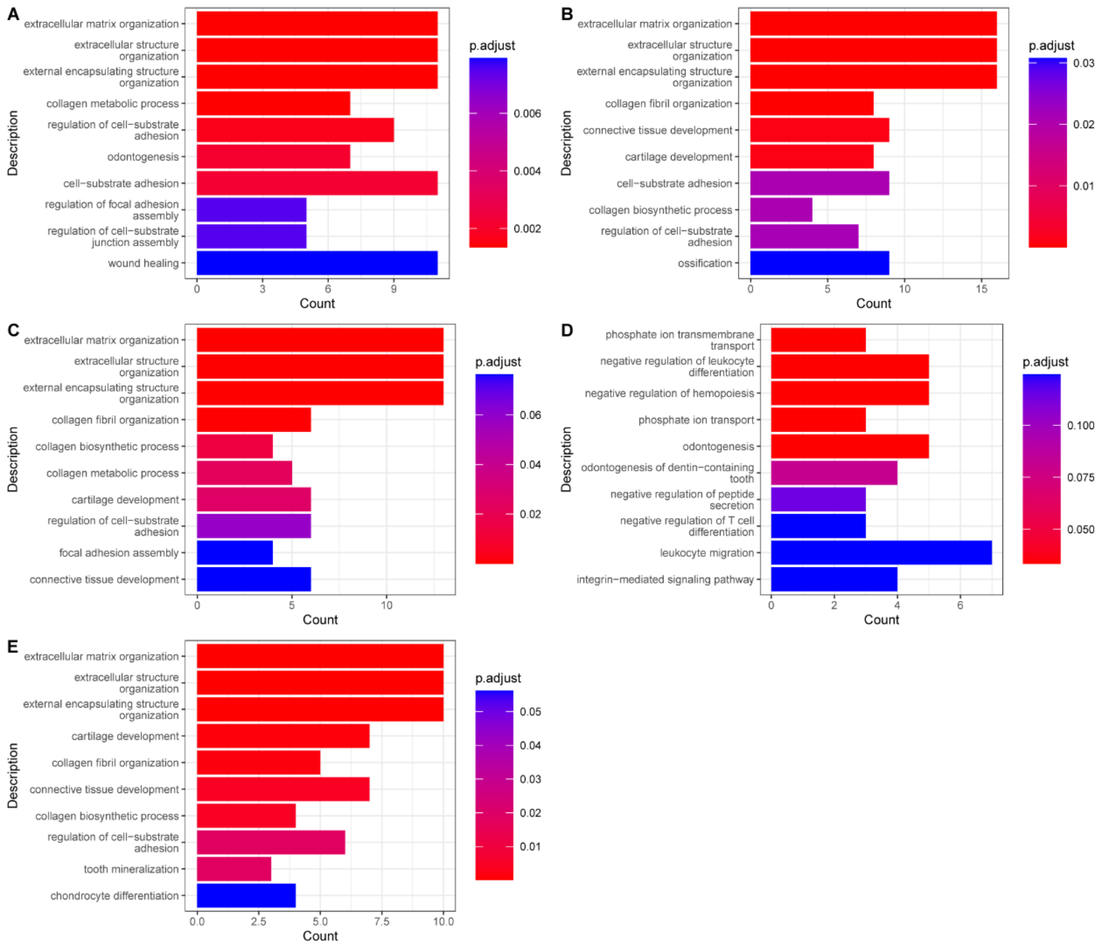

Figure S1. GO enrichment of mRNA trans-regulated by hub lncRNAs

GO enrichment analysis for mRNA trans-regulated by **A.** *MIR4435-2HG*; **B.** *FAM225A*; **C.** *BHLHE40-AS1*; **D.** *LINC01614*; **E.** *AL356417.2*.
