# Supplementary material for: Differential expression profiles and functional analysis of long non-coding RNAs in calcific aortic valve disease
Source: BMC Cardiovasc Disord. 2023 Jun 27;23:326. doi: 10.1186/s12872-023-03311-x (PMC10294343; doi:10.1186/s12872-023-03311-x)
Supplement: Supplementary file 2 — Supplementary Material 2 [file 12872_2023_3311_MOESM2_ESM.pdf]

Differential Expression Profiles and Functional Analysis of Long Non-coding RNAs in Calcific Aortic Valve Disease

Guang-Yuan Song[1]*,Xu-Nan Guo[1],Jing Yao[1],Zhi-Nan Lu[1],Jia-Hong Xie[1],Fang wu[2],Jing He[1],Zhao-Lin Fu[1],Jie Han[2]*

[1]Interventional Center of Valvular Heart Disease, Beijing Anzhen Hospital Affiliated to Capital Medical University, Beijing, China

[2]Department of Cardiac Surgery, Beijing Anzhen Hospital Affiliated to Capital Medical University, Beijing, China

* Co-Correspondence author: Guang-Yuan Song, email: songgy_anzhen@vip.163.com, Jie Han, email:drhanjie@163.com

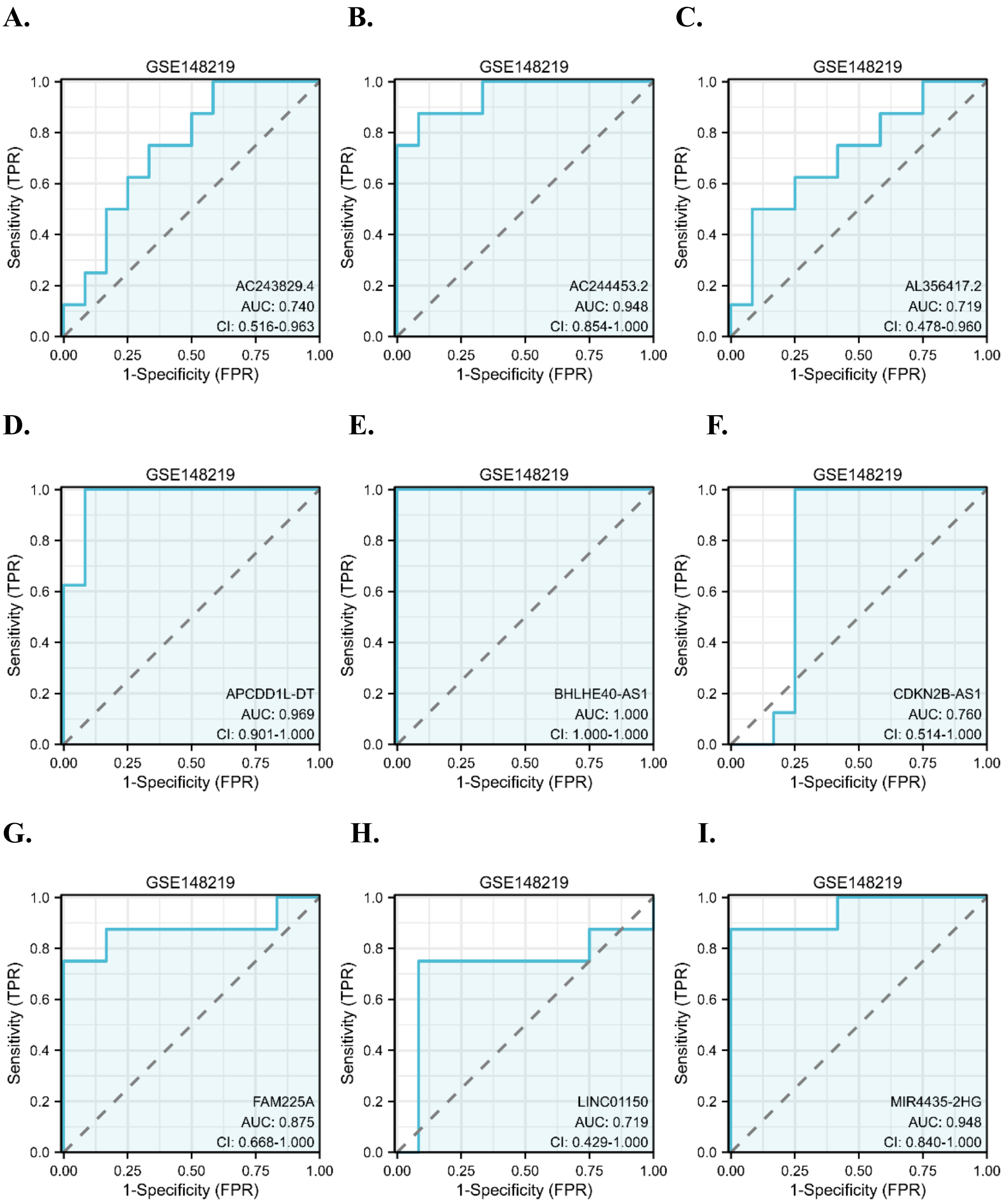

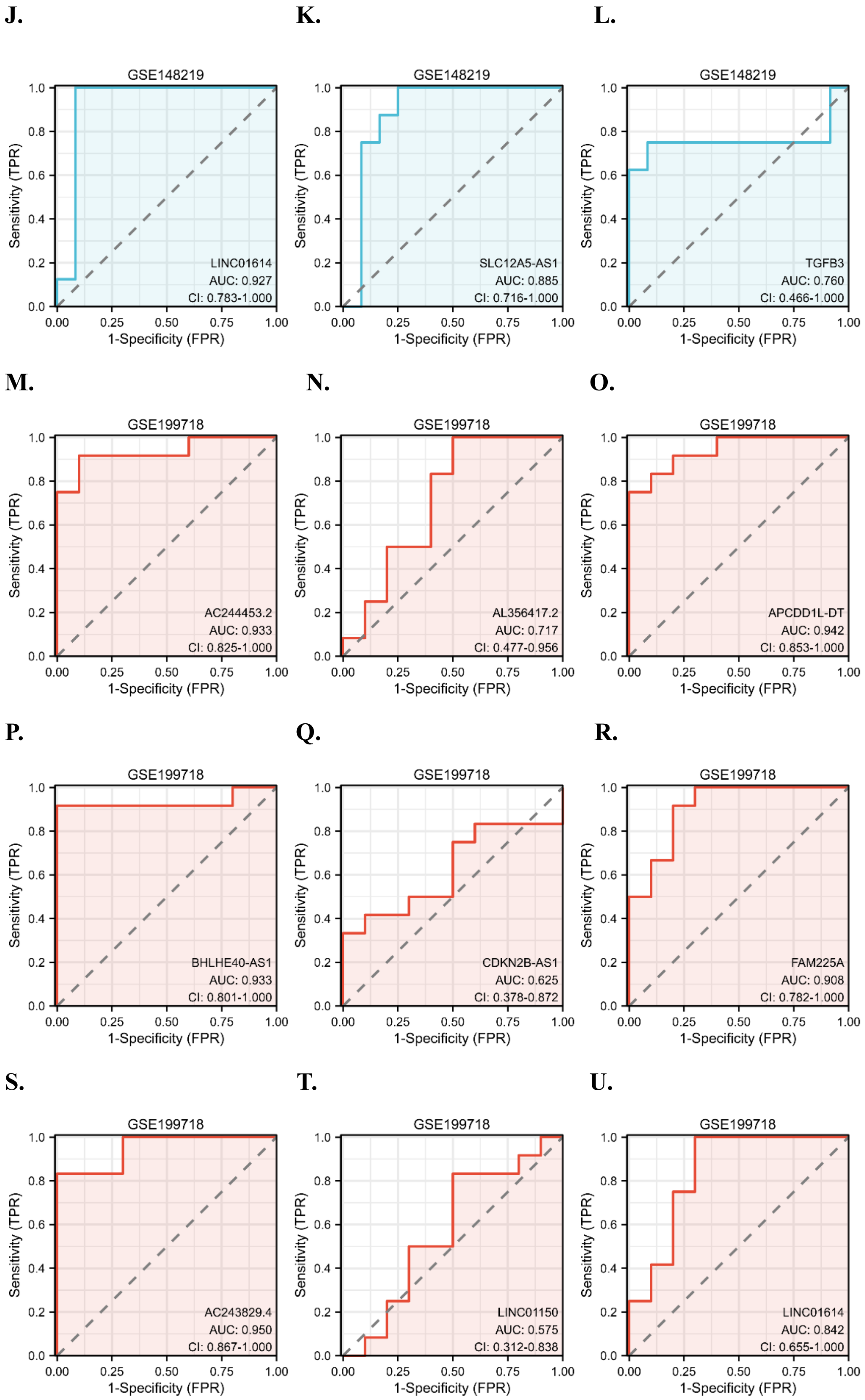
J.
GSE148219
LINC01614
AUC: 0.927
CI: 0.783-1.000
K.
GSE148219
SLC12A5-AS1
AUC: 0.885
CI: 0.716-1.000
L.
GSE148219
TGFB3
AUC: 0.760
CI: 0.466-1.000
M.
GSE199718
AC244453.2
AUC: 0.933
CI: 0.825-1.000
N.
GSE199718
AL356417.2
AUC: 0.717
CI: 0.477-0.956
O.
GSE199718
APCDD1L-DT
AUC: 0.942
CI: 0.853-1.000
P.
GSE199718
BHLHE40-AS1
AUC: 0.933
CI: 0.801-1.000
Q.
GSE199718
CDKN2B-AS1
AUC: 0.625
CI: 0.378-0.872
R.
GSE199718
FAM225A
AUC: 0.908
CI: 0.782-1.000
S.
GSE199718
AC243829.4
AUC: 0.950
CI: 0.867-1.000
T.
GSE199718
LINC01150
AUC: 0.575
CI: 0.312-0.838
U.
GSE199718
LINC01614
AUC: 0.842
CI: 0.655-1.000
Sensitivity (TPR)
1-Specificity (FPR)
0.0
0.2
0.4
0.6
0.8
1.0
0.00
0.25
0.50
0.75
1.00

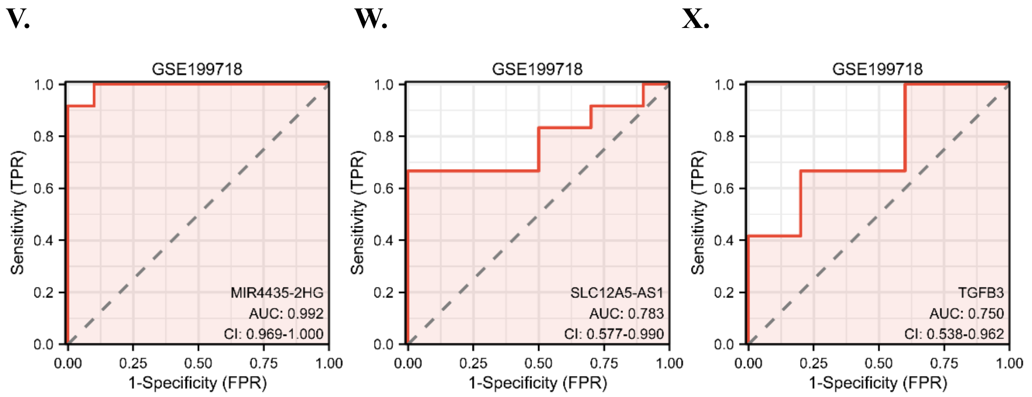

Figure S2 : ROC curves analysis of hub cis- or trans-regulatory genes and GSE199718

ROC curves analysis of hub cis- or trans-regulatory genes **A.** *AC243829.4* in GSE148219*;* **B.** *AC244453.2* in GSE148219; **C.** *AL356417.2* in GSE148219; **D.** *APCDD1L-DT* in GSE148219**;** **E.** *BHLHE40-AS1* in GSE148219; **F.** *CDKN2B-AS1* in GSE148219; **G.** *FAM225A* in GSE148219*;* **H.** *LINC01150* in GSE148219; **I.** *MIR4435-2HG* in GSE148219; **J.** *LINC01614* in GSE148219*;* **K.** *SLC12A5-AS1* in GSE148219; **L.** *TGFB3* in GSE148219; **M.** *AC244453.2* in GSE199718; **N.** *AL356417.2* in GSE199718; **O.** *APCDD1L-DT* in GSE199718; **P.** *BHLHE40-AS1* in GSE199718; **Q.** *CDKN2B-AS1* in GSE199718; **R.** *FAM225A* in GSE199718; **S.** *AC243829.4* in GSE199718; **T.** *LINC01150* in GSE199718; **U.** *LINC01614* in GSE199718; **V.** *MIR4435-2HG* in GSE199718; **W**. *SLC12A5-AS1* in GSE199718; **X.** *TGFB3* in GSE199718;
